# *VEGFA* and *NFE2L2* Gene Expression and Regulation by MicroRNAs in Thyroid Papillary Cancer and Colloid Goiter

**DOI:** 10.3390/genes11090954

**Published:** 2020-08-19

**Authors:** Leonardo P. Stuchi, Márcia Maria U. Castanhole-Nunes, Nathália Maniezzo-Stuchi, Patrícia M. Biselli-Chicote, Tiago Henrique, João Armando Padovani Neto, Dalisio de-Santi Neto, Ana Paula Girol, Erika C. Pavarino, Eny Maria Goloni-Bertollo

**Affiliations:** 1 Research Unit in Genetics and Molecular Biology—UPGEM, Faculty of Medicine of São José do Rio Preto—FAMERP, São José do Rio Preto 15090-000, Brazil; prado_leonardo@yahoo.com.br (L.P.S.); mcastanhole@gmail.com (M.M.U.C.-N.); patriciabiselli@yahoo.com.br (P.M.B.-C.); erika@famerp.br (E.C.P.); 2 Padre Albino University Center—UNIFIPA, Catanduva, São Paulo 15809-144, Brazil; nmmbiomedica@hotmail.com (N.M.-S.); anapaula.girol@unifipa.com.br (A.P.G.); 3 Laboratory of Molecular Markers and Bioinformatics, Department of Molecular Biology, Faculty of Medicine of São José do Rio Preto —FAMERP, São José do Rio Preto 15090-000, Brazil; henrique@famerp.br; 4 Department of Otolaryngology and Head and Neck Surgery, Faculty of Medicine of São José do Rio Preto —FAMERP, São José do Rio Preto 15090-000, Brazil; padovani.ja@gmail.com; 5 Pathological Anatomy Service, Hospital de Base, Foundation Regional Faculty of Medicine of São José do Rio Preto—FUNFARME, São José do Rio Preto 15090-000, Brazil; dalisius@gmail.com

**Keywords:** thyroid neoplasms, goiter, vascular endothelial growth factor A, NF-E2-related factor 2, microRNAs

## Abstract

Deregulation of VEGFA (Vascular Endothelial Growth Factor A) and NFE2L2 (Nuclear Factor (Erythroid-derived 2)-Like 2), involved in angiogenesis and oxidative stress, can lead to thyroid cancer progression. MiR-17-5p and miR-612 are possible regulators of these genes and may promote thyroid disorders. In order to evaluate the involvement of VEGFA, NFE2L2, hsa-miR-17-5p, and hsa-miR-612 in thyroid pathology, we examined tissue samples from colloid goiter, papillary thyroid cancer (PTC), and a normal thyroid. We found higher levels of VEGFA and NFE2L2 transcripts and the VEGFA protein in goiter and PTC samples than in normal tissue. In the goiter, miR-612 and miR-17-5p levels were lower than those in PTC. Tumors, despite showing lower *VEGFA* mRNA expression, presented higher VEGFA protein levels compared to goiter tissue. In addition, NRF2 (Nuclear Related Transcription Factor 2) protein levels in tumors were higher than those in goiter and normal tissues. Inhibition of miR-17-5p resulted in reduced NFE2L2 expression. Overall, both transcript and protein levels of NFE2L2 and VEGFA were elevated in PTC and colloid goiter. Hsa-miR-612 showed differential expression in PTC and colloid goiter, while hsa-miR-17-5p showed differential expression only in colloid goiter, suggesting that hsa-miR-17-5p may be a positive regulator of NFE2L2 expression in PTC.

## 1. Introduction

Colloid goiter is the most common disorder of the thyroid gland, even in non-endemic regions, and it is clinically detected in about 4% of individuals older than 30 years [1]. The presence of colloid goiter can indicate the beginning of malignant transformation of the thyroid leading to thyroid cancer [2,3].

Thyroid cancer is the most common endocrine neoplasia, accounting for about 1.7% of all cancer diagnoses worldwide [4], and it is the fifth most common type of cancer in women [5]. Papillary thyroid cancer (PTC) is the most common thyroid cancer, accounting for about 80% of diagnoses [6]. 

Angiogenesis plays a key role in the progression of cancer and the onset of metastases, as the newly formed blood vessels supply the nutrients and oxygen necessary for the maintenance of tumor growth [7]. VEGFA (Vascular Endothelial Growth Factor A) is the first angiogenic factor induced by hypoxia and promotes proliferation, budding, migration, and formation of the endothelial matrix [8]. Another important factor for angiogenesis is nuclear related transcription factor 2 (NRF2), encoded by the *NFE2L2* gene (*nuclear factor (erythroid-derived 2)-like 2*). NRF2 regulates the expression of antioxidant proteins in response to oxidative stress in various tissues [9]. Therefore, *VEGFA* and *NFE2L2* have been considered potential targets for new antiangiogenic therapies.

Gene expression can be regulated by microRNAs (miRNAs, miR), which control many cellular processes, including cell growth, differentiation, proliferation, and apoptosis [10]. Identification of the possible roles of miR-17-5p and miR-612 in the regulation of *NFE2L2* and *VEGFA* expression in PTC and colloid goiter can provide valuable insights for the development of strategies and drugs to inhibit tumor growth and also to restore sensitivity of tumors to chemotherapy.

In this study, we aimed to evaluate mRNA and protein levels of VEGFA and NFE2L2, as well as the expression patterns of miR-17-5p and miR-612 in human papillary thyroid cancer, colloid goiter, and normal thyroid tissues, and also to investigate the involvement of miR-17-5p and miR-612 in the regulation of *VEGFA* and *NFE2L2* expression in the thyroid papillary cancer cell line (TPC-1 line).

## 2. Materials and Methods

### 2.1. Specimens

Tumor and goiter tissue samples, along with adjacent tissues, as well as normal thyroid tissue samples were collected from 66 patients, as follows: 15 thyroid papillary cancer patients (13 females and 2 males), 15 goiter colloid patients (14 females and 1 male), and 6 patients with normal thyroid (4 females and 2 males). Tumor and goiter samples, along with adjacent tissue samples, were sent to the Pathology Service of Hospital de Base de São José do Rio Preto—SP for diagnosis and microdissection. The tumors were classified according to the parameters of “American Joint Committee for Cancer” (AJCC) [11]: tumor size (T), presence of nodal metastasis (N), and presence of distant metastasis (M). This study was approved by the Research Ethics Committee of the Medical School of São José do Rio Preto, FAMERP (No. 468.393). 

### 2.2. Computer Prediction of miRs

miRs were selected in the DIANA-TarBase v7.0 database (http://diana.imis.athena-innovation.gr/DianaTools/index.php?r=tarbase/index), TargetScan (http://www.targetscan.org/vert_71) and mirDIP (http://ophid.utoronto.ca/mirDIP/). Two miRs with the highest score for regulation of *NFE2L2* and *VEGFA* were selected.

### 2.3. Expression of NFE2L2, VEGFA, miR-17-5p, and miR-612

RNA was extracted using the mirVana PARIS Kit (Applied Biosystems, Carlsbad, CA, USA). Complementary DNA (cDNA) from total RNA was synthesized using the High Capacity cDNA Archive Kit (Life Technologies, Carlsbad, CA, USA). The conversion of the miRs into cDNA was performed using the TaqMan-Micro RNA Reverse Transcription kit (Applied Biosystems).

Expression analyses of *NFE2L2* (Hs00975961_g1) and *VEGFA* (Hs00900055_m1), miR-17-5p (002308), and miR-612 (001579) were performed by quantitative real-time PCR (qPCR) using specific TaqMan probes (Thermo Fisher Scientific, Waltham, MA, USA) on the CFX 96 Real Time System (Bio-Rad, Hercules, CA, USA). All reactions were performed in duplicates and included a contamination control. The genes *β-actin* (Hs01060665_g1) and *GAPDH* (Hs03929097_g1) were used as reference genes for normalization of *NFE2L2* and *VEGFA* expression data. The genes *RNU6B* (001093) and *RNU48B* (001006) were used for normalization of miR-17-5p and miR-612 expression data (Thermo Fisher Scientific). Relative quantification (RQ) of genes and miR expression in PTC and colloid goiter was calculated using the 2^-ΔΔCt^ method in relation to the normal tissues [12]. 

### 2.4. Quantification of Protein Expression in Tissue Samples

The proteins were extracted using the mirVana Paris Kit and Trizol Reagent (Applied Biosystems) and quantified using the BCA Protein Assay Kit (Abcam, Cambridge, United Kingdom).

Quantification of VEGFA protein in fresh tissue samples was performed using VEGFA Duo Set ELISA Kit (R&D Systems, Minneapolis, MN, USA) following the manufacturer’s instruction. Immunohistochemistry was performed for analysis of NRF2 protein quantification. Briefly, after deparaffinization, the sections were rehydrated in a graded series of ethanol. The polyclonal rabbit anti-Nrf2 primary antibody (PA5-27882, Thermo Fisher Scientific) was used at a dilution of 1:100. After incubation, a biotinylated secondary antibody (Histostain-Plus IHC Kit, DAB, broad spectrum, 95-9943B, Invitrogen, Carlsbad, CA, USA) was used. The slides were incubated with streptavidin complex conjugated to peroxidase and 3,3′-diaminobenzidine (DAB 750118, Invitrogen) in the dark. For analysis of densitometry, the sections were photographed under a 40x objective (three fields per slide). For each sample, the cytoplasm and nucleus of epithelial cells were evaluated at 20 points equally distributed in the cytoplasm and 10 points in the nucleus.

### 2.5. Cell Line TPC-1 Culture

The TPC-1 cell line [13] derived from female papillary cancer was cultured in DMEM (Dulbecco’s modified Eagle’s medium, Cultilab, Campinas, Brazil), supplemented with 10% fetal bovine serum (Cultilab), 100 U/mL sodium penicillin, 100 mg/mL streptomycin (Cultilab), and 1% l-glutamine (Cultilab) at 37 °C in a 5% CO_2_ incubator. The TPC-1 cell line authentication was performed by STR (Short Tandem Repeat) DNA typing profile using Gene Print 10 (Promega, Madison, WI, USA), ID 142738.

### 2.6. Transfection in the TPC-1 Cell Line

Transfection assays were conducted using mirVana™ inhibitor for miR-17-5p (MH12412, Thermo Scientific) and the mirVana™ miR-612 mimic (MC11461, Thermo Scientific) with Lipofectamine RNAiMAX (Invitrogen) following the manufacturer’s instructions. Cells were cultured for 48 h in 100 μL of Opti-MEM serum-free medium (Invitrogen), 1 μL of Lipofectamine RNAiMAX (Invitrogen), and 10 mM of the inhibitor for miR-17-5p or the miR-612 mimic. RNA was extracted to verify the efficiency of transfection, using the respective positive and negative controls by qPCR.

### 2.7. Statistical Analyses

Statistical analyses were performed using GraphPad Prism software, version 6. The continuous data distribution was evaluated using D’Agostino and Pearson’s normality test. The Wilcoxon signed rank test and the Mann–Whitney test were used to evaluate the gene expression data. The correlation between the expression of miRNAs and the genes was analyzed by Spearman’s correlation. The Mann–Whitney test was used to evaluate the protein expression data. Values of *p* < 0.05 were considered significant. 

## 3. Results

### 3.1. Characteristics of the Samples

The characteristics of the samples are summarized in Table 1.

### 3.2. Expression of VEGFA, NFE2L2, miR-17-5p, and miR-612 in Fresh Tissue Samples

Expression levels of *VEGFA, NFE2L2*, miR-17-5p, and miR-612 in the tumor tissues, colloid goiter, and their respective adjacent tissues were compared to those observed in the normal tissues. *VEGFA* and *NFE2L2* showed high expression levels in the tumor and goiter. MiR-17-5p and miR-612 did not exhibit differential expression in the tumor, but the expression levels of both miRs were reduced in the goiter (Table 2).

*VEGFA* and *NFE2L2* also showed elevated expression in the tumor- and goiter-adjacent tissues. MiR-612 showed reduced expression in the tumor-adjacent tissue and in the goiter-adjacent tissue, whereas miR-17-5p showed reduced expression only in the goiter-adjacent tissue (Table 3).

**Figure 1:**
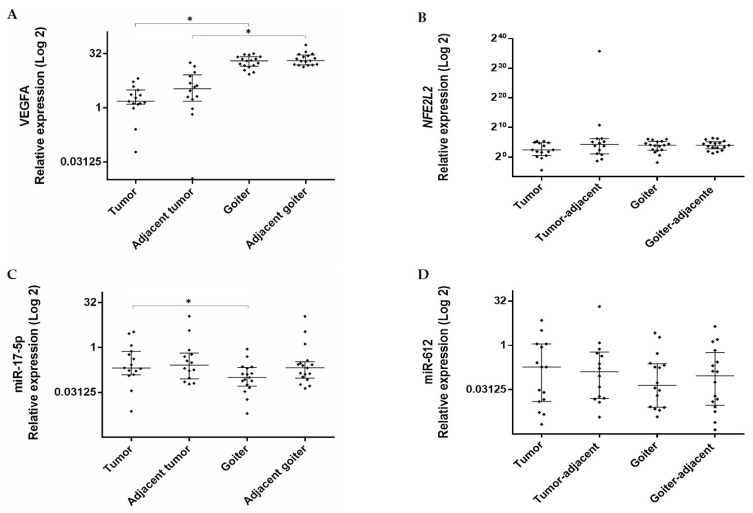
Expression levels of (**A**) *VEGFA* (*Vascular Endothelial Growth Factor A*), (**B**) *NFE2L2 (Nuclear Factor (Erythroid-derived 2)-Like 2*), (**C**) miR-17-5p, and (**D**) miR-612 in tumor and goiter tissues and their respective adjacent tissues. Data are presented as median with interquartile range (25% percentile and 75% percentile). The relative expression value was Log2 transformed (*y*-axis). Calibrator (normal tissue) log RQ = 1. *, Statistically significant (panel A, Mann–Whitney, *p* < 0.0001; panel C, Mann–Whitney, *p* = 0.033).

Comparisons between the groups revealed that *VEGFA* gene expression was higher in the goiter than in the tumor (RQ median = 20.28 vs. 1.5; *p* < 0.0001) and also in the goiter-adjacent tissue than in the tumor-adjacent tissue (RQ median = 20.72 vs. 3.40, *p* < 0.0001) (Figure 1). No significant difference was detected in *NFE2L2* expression between the groups.

Regarding miR expression, miR-17-5p expression was higher in the tumor than in the goiter (RQ median = 0.20 vs. 0.09; *p* = 0.033) (Figure 1). Expression of miR-612 did not differ significantly between the groups.

### 3.3. Correlation between Expression Levels of VEGFA, NFE2L2, miR-17-5p, and miR-612

**Figure 2:**
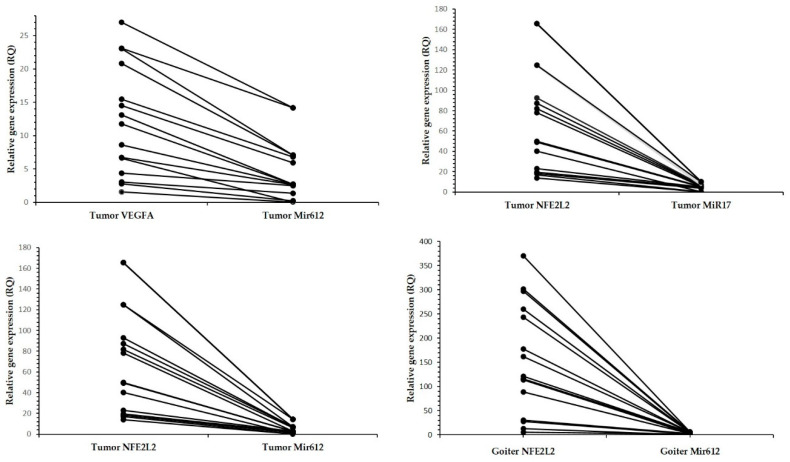
Relationship between relative expression levels of miRs and genes, showing high expression levels of genes and low expression levels of miRs. Each point represents an individual sample, for which the two gene expression levels are correspondingly connected. RQ, relative quantification.

There was a negative correlation in the tumor tissue between miR-612 and *VEGFA* expression, and between miR-612 and miR-17-5p and *NFE2L2* expression. In relation to the goiter, only miR-612 expression presented a negative correlation with *NFE2L2* expression (Table 4; Figure 2).

### 3.4. Expression of VEGFA and NRF2 Proteins in Tissues

**Figure 3:**
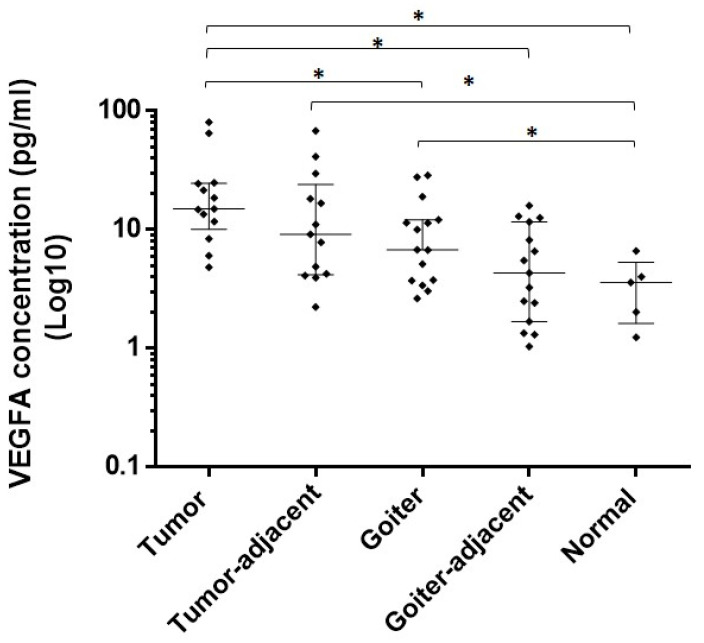
Protein expression of VEGFA in tumor, goiter, and normal tissue samples. VEGFA concentration was Log10 transformed (*y*-axis). Mann–Whitney test (* *p* value).

The protein levels of VEGFA were higher in the tumor compared to those in normal tissue *(p* = 0.0009), the goiter (*p* = 0.0222), and goiter-adjacent tissue (*p* = 0.0003). Tumor-adjacent tissue also presented elevated VEGFA protein levels compared to the normal tissues (*p* = 0.0138). The expression of VEGFA was upregulated in the goiter compared to the normal tissues (*p* = 0.0397) (Figure 3). 

**Figure 4:**
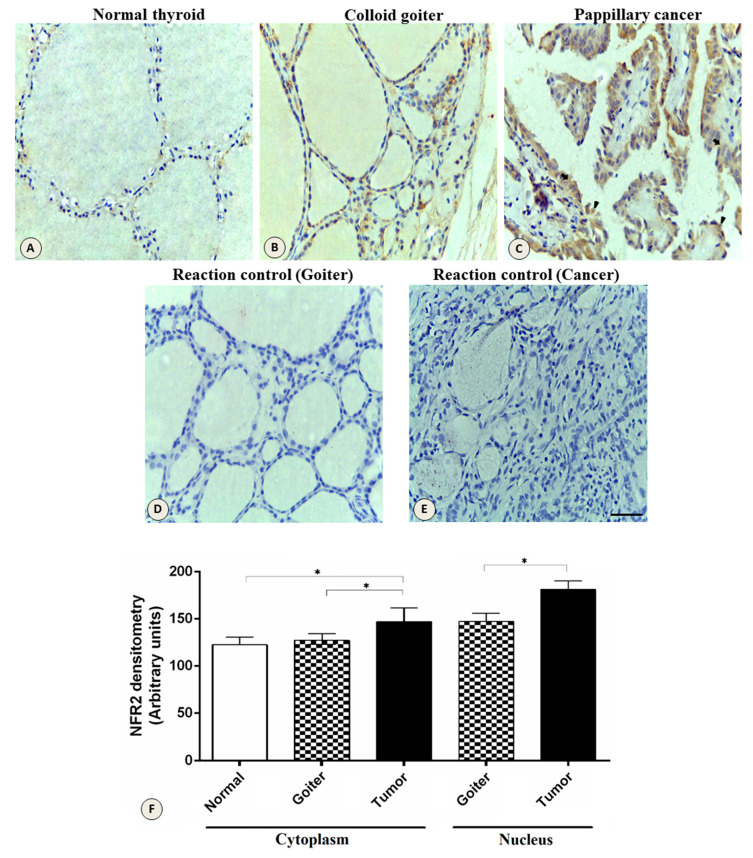
Expression of the NRF2 protein in the thyroid gland. Increased expression in the cytoplasm and nucleus in different tissues. (**A**) Normal thyroid tissue. (**B**) Colloid goiter. (**C**) Papillary thyroid cancer. (**D**) Negative controls of the goiter and (**E**) tumor. Section thickness, 5 μm; contra-staining, hematoxylin; scale bars, 20 μm. (**F**) Densitometric analysis of NRF2 quantification. The data represent the mean ± SEM of the densitometric index. *, *p* < 0.0001; bar size, 20 µm; arrows, cytoplasm; arrowhead, nucleus.

Expression of NRF2 protein in tumor tissues, colloid goiter, and normal tissues is shown in Figure 4. The cytoplasmic expression of NRF2 was higher in the tumor tissues compared to the normal tissues (*p* < 0.0001) and the goiter *(p* < 0.0001). In the nucleus, there was a stronger staining in the tumor tissue compared to the goiter tissue (*p* <0.0001); no nuclear staining was observed in the normal thyroid tissues.

### 3.5. Superexpression Assay of miR-612 in the TPC-1 Cell Line

The transfection efficiency was checked on TPC-1 cells using the positive (mirVana™ miRNA Mimic miR-1 Positive Control, Life Technologies) and negative controls (mirVana ™ miRNA Mimic, Negative Control # 1, Life Technologies). Relative quantification (2-ΔΔCt method) of *TWF1* in cells treated with mirVana ™ miRNA Mimic miR-1 Positive Control revealed a 60% reduction in *TWF1* expression. The transfection with miR-612 did not show any significant difference in the expression of *VEGFA* and *NFE2L2* in the treated cells.

### 3.6. Inhibition Assay of miR-17-5p in the TPC-1 Cell Line

**Figure 5:**
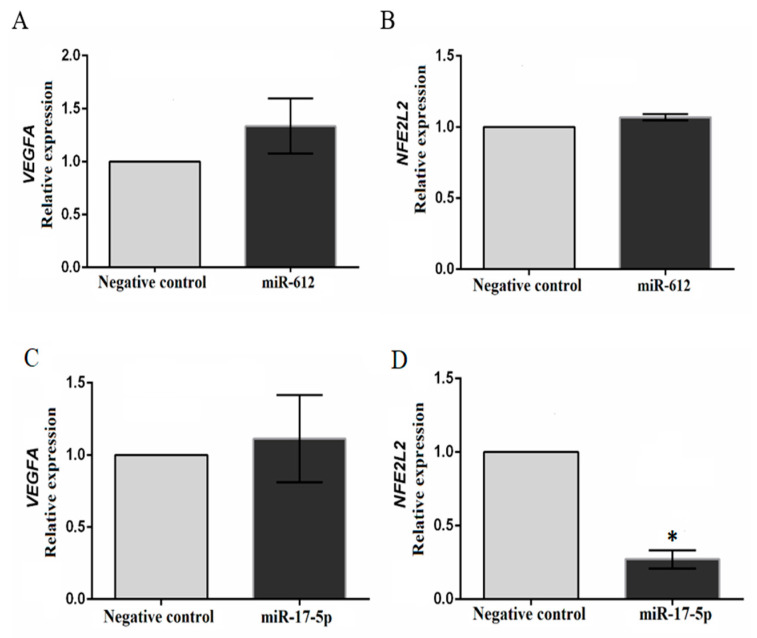
Results of the transfection assay on the TPC-1 cell line using mirVana™ miRNA Inhibitor for miR-17-5p, and mirVana™ miRNA Mimic for miR-612. *VEGFA* expression did not differ between cells transfected and non-transfected with miR-612 (**A**) and the inhibitor for miR-17-5p (**C**). *NFE2L2* expression did not differ between cells transfected and non-transfected with miR-612 (**B**); the inhibition of miR-17-5p resulted in approximately 73% inhibition of *NFE2L2* expression (**D**). *, approximately 73% inhibition of gene expression in relation to the negative control. Calibrator (negative control) log RQ = 1.

The transfection efficiency test for the inhibition assay using the positive control mirVana™ miRNA Inhibitor, let-7c positive control (Life Technologies) showed a 60% reduction in *HMGA2* expression. Transfection of miR-17-5p inhibitor into TPC-1 cells showed no difference in *VEGFA* expression, however, an approximately 73% inhibition in *NFE2L2* expression was noted (Figure 5). 

## 4. Discussion

We noted higher prevalence of cancer and goiter in females, which may be related to sex-differences. Porc et al. reported the first meta-analysis and revealed genetic factors that differentially affect thyroid function in males and females [14]. The present study shows that tissues affected by papillary thyroid cancer or colloid goiter, and their respective adjacent tissues present increased expression of *VEGFA* and *NFE2L2*, thereby providing evidence that vascularization and oxidative stress are imbalanced in these tissues. Corroborating with our results, earlier studies have shown that the expression of the *VEGFA* is higher in thyroid cancer, and may be crucial for the development of goiter, since the proliferation of endothelial cells precedes that of thyroid cells [15,16,17,18]. Studies have reported functional defects and increased expression of *NFE2L2* in the thyroid. Since this gene is involved in the maintenance of homeostasis against the physiologically generated oxidative stress during thyroid hormone synthesis, its dysregulation may contribute to the development of goiter as well as tumorigenesis of thyroid [19,20,21]. It is noteworthy that the expression levels of these genes increase according to the type and degree of thyroid cancer progression [15,22].

We also observed the elevated expression of these genes in the tissues adjacent to the tumor tissues in relation to the normal tissues. This indicates that the organ affected by cancer and goiter present altered physiology. This result highlights the importance of the normal tissue as a normalizer of the expression data in gene expression studies, since the tissue adjacent to tumor may not represent the normality of the tissue in true sense and, in fact, may present some of cellular alterations that precede these diseases. To the best of our knowledge, this is the first study to analyze the expression of *VEGFA* and *NFE2L2* in normal thyroid tissue for comparison with tumor tissue and goiter, as other studies have used adjacent non-malignant tissues for comparison.

The TCGA (The Cancer Genome Atlas Program) thyroid cancer dataset showed that VEGFA and NFE2L2 were highly expressed in normal tissue (*n* = 59) when compared with the primary tumor (*n* = 505). When analyzing the histology of thyroid tumors, it was found that, for VEGFA, the follicular thyroid papillary carcinoma (*n* = 102) was the closest to normal tissue, while the classical thyroid papillary carcinoma (*n* = 358), tall thyroid papillary carcinoma (*n* = 36) and others (*n* = 9) were down-regulated in relation to normal tissue. All NFE2L2 levels in cancer tissues were down-regulated in relation to normal tissue (Supplementary Material). These changes in expression levels in relation to normal tissue may be related to the sample number analyzed.

A comparison between tumor and colloid goiter and between tumor-adjacent tissue and goiter-adjacent tissue revealed a significantly higher expression of the *VEGFA* gene in the samples of colloid goiter and its adjacent tissue. This increase in the expression of the *VEGFA* gene in goiter may be due to several factors. Increasing endothelial cells is the first step in goiter formation, which exhibits levels of *VEGFA* expression similar to those observed in some types of thyroid cancer [23,24,25]. It is also worth mentioning that most of the papillary cancer samples analyzed in the present study presented early stages according to the TNM (Classification of Malignant Tumors) classification and that the levels of *VEGFA* gene expression increased according to the types and stages of tumor [15].

The expression of the *NFE2L2* gene did not differ significantly between the tissues. Evidence suggests that the difference between the tissues can be more effectively analyzed with the protein analysis of NRF2, because this protein occurs in degraded form in the normal tissue. In benign lesions, this degradation undergoes an imbalance, whereas in the tumor tissue it is possible to observe the migration of NRF2 to the nucleus, where this factor acts in response to oxidative stress, thus activating antioxidant enzymes [20,22].

In addition, the expression of miR-17-5p and miR-612 in PTC and colloid goiter samples and their respective adjacent tissues was also verified in comparison to the normal tissues, since these miRs may be related to the regulation of the genes investigated in this study. The results showed that miR-17-5p and miR-612 were not differentially expressed in tumor samples, and only miR-612 expression was reduced in tumor-adjacent tissue. In colloid goiter samples and goiter-adjacent tissue, the expression of these miRs was reduced.

MiR-17-5p has been studied in several cancers types, including thyroid cancer, and the results point to an elevated expression in tumor tissues [26,27,28]. However, the data available for PTC are not consistent with our findings. Zhao and Li (2015) observed significantly reduced miR-17-5p expression in thyroid papillary cancer samples in comparison to tumor-adjacent tissues [29]. The present study, however, did not find significant differences between the expression levels of miR-17-5p in tumor and non-tumor adjacent tissues, but a slight decrease in expression was observed; a larger sample size is required to reach a conclusion. Expression levels of miR-17-5p, on the other hand, were significantly reduced in the colloid goiter samples compared to those in normal tissues. No reports were found in the literature that corroborate or contrast with the findings of the present study. In the comparison between the tumor and goiter samples, we detected a significant increase in miR-17-5p expression in the tumor samples, indicating that even in initial stages the malignant tissue presents alteration of expression in comparison to the benign tissue.

Expression of miR-612 showed no significant difference in the tumor tissues, although its relative expression was low in these samples. However, in the tumor-adjacent tissues, colloid goiter and goiter-adjacent tissues, the expression of miR-612 was significantly reduced compared to that in the normal tissues. It was previously shown that this miR-612 exhibits reduced expression in colorectal cancer [30] and hepatocellular carcinoma (HCC), and that its expression is inversely proportional to tumor progression and aggressiveness in HCC [31]. Our results, deviating from the observations in HCC and colorectal cancer, suggest that the expression of this miR could increase with the degree of cellular alterations that lead to tumor formation, since the tumor-adjacent tissues and the goiter samples have significantly reduced expression of miR-612. As no data on miR-612 expression in PTC are available, further investigations are needed to clarify the role of miR-612 in this and other types of thyroid disorders. 

The protein quantification results of VEGFA showed that the papillary cancer samples, the tumor-adjacent tissues, and the colloid goiter samples had increased expression of this protein in comparison to the normal tissues. In the comparisons between goiter and tissues adjacent to the goiter as well as between tumor tissues and tumor-adjacent tissues, no difference in VEGFA protein or transcript levels was observed. A comparison of VEGFA levels between tumor and goiter samples showed significantly higher expression in the tumor, a result suggesting a relation at the protein level opposite to that we observed in mRNA levels. This contradictory result between protein and transcript levels suggests that tumor tissue and goiter may present different mechanisms of post-transcriptional or post-translational regulation that could result in lower amounts of protein present in goiter. 

In addition, VEGFA also participates in angiogenic stimulation through inhibition of PTEN (phosphatase and tensin homolog) expression. VEGFA triggers a cascade of signaling events, including activation of mitogen-activated protein kinase (MAPK) and phosphorylation of the Elk-1 transcription factor. These events promote an increase in the expression of members of the miR-17-92 group, repressing PTEN expression. It is known that the reduced expression of PTEN protein is associated with the development of thyroid cancer through the proliferation of endothelial cells. On the contrary, the genetic inactivation of miR-17-92 in endothelial cells results in peripheral vascular impairment in vivo [32,33]. These findings reinforce the importance of increased VEGFA protein in tumors and may explain the higher protein quantification observed in the tumor samples in comparison to the goiter.

Using the immunohistochemistry technique, it was possible to observe the presence of NRF2 protein, which is activated by oxidative stress, migrating from the cell cytoplasm to the nucleus where it exerts the function of transcription factor. The results observed in the present study showed that the labeling of this protein in the normal tissue is very weak (almost undetectable), and is observed only in the cytoplasm. In goiter, the labeling is intensified and it is already possible, in some cases, to observe the protein in the nucleus. Finally, in the tumor tissue, it is possible to see a strong labeling in the cytoplasm and nucleus. These data indicate that NRF2 protein is activated in PTC. Consistent with our data, Ziros et al. (2013) also observed the same labeling pattern in normal thyroid tissue, benign lesions and papillary cancer [20]. A recent study by Geng et al. (2017) indicates that the Nrf2 pathway is commonly activated in PTC and occasionally is activated in benign thyroid lesions, suggesting that prolonged activation of NRF2 and its elevated expression may contribute to the occurrence of nodular goiter and PTC [22]. The presence of strong labeling of the NRF2 protein in the nucleus of tumor cells reinforces its role in the regulation of antioxidant response related genes [34].

To obtain information about the possible miRs involved in the regulation of the *NFE2L2* and *VEGFA* genes, a database search was performed. Bioinformatic analysis revealed that miR-17-5p targets both *VEGFA* and *NFE2L2* genes. Previous studies have shown the interaction between miR-17 and *VEGFA* in the skin [35] and kidney [36,37]. The interaction of this miR with the *NFE2L2* gene was observed in the mammary gland [38]. The miR-612 showed regular expression of the *VEGFA* gene in the bone marrow [39]. To date, there are no studies that prove the interaction of miR-612 with *NFE2L2;* however, according to the databases consulted, miR-612 is predicted to regulate the *NFE2L2* gene. 

After bioinformatic analysis, the inhibitor for miR-17-5p and the miR-612 mimic were selected for transfection into the TPC-1 cell line. The present study showed that inhibition of miR-17-5p resulted in reduced expression of the *NFE2L2* gene in the TPC-1 cells. The miR-17 cluster is known to reduce the expression of the *PTEN* gene. The *PTEN* gene is important for angiogenesis as it regulates genes such as *NFE2L2*. Rojo et al. (2014) showed that the inhibition of miR-17 increased *PTEN* expression, which in turn has a role in Nrf2 degradation pathway [40]. In the present study inhibition of miR-17-5p resulted in a decrease in *NFE2L2* expression, corroborating previous findings; however, the tissue expression data showed a negative correlation between the expression of this miR-17-5p and the *NFE2L2* gene. These contrasting results may reflect the participation of other active pathways in thyroid cancer that could influence *NFE2L2* expression. It is also worth mentioning the differences found between the findings of fresh tumor tissue samples and cell culture. Although in vitro studies are extremely important for functional studies, they cannot reproduce the complexity of the tumor microenvironment, nor can they mimic the different pathways involved in the disease progression. 

The genes and miRs evaluated in this study present great potential for the diagnosis of colloid goiter and papillary cancer since they presented differential expression in these tissues. VEGFA and NRF2 proteins have been shown to be efficient in differentiating normal tissues from PTC. The negative regulation of miR-17-5p and miR-612 in colloid goiter also suggest the performance of these miRs as biomarkers for this thyroid condition. 

Angiogenesis, oxidative stress, and deregulation of miRs are crucial for the development of thyroid disorders and cancer. The two genes evaluated in the present study can be possible therapeutic targets in the angiogenesis of thyroid disorders since the feedback between these genes and their regulation via miRs is of great importance for tumor development. 

## 5. Conclusions

*NFE2L2* and *VEGFA* genes and their protein products are widely expressed in PTC and colloid goiter. miR-612 has differential expression in the thyroid tumor and colloid goiter, while miR-17 is expressed only in the goiter. Hsa-miR-17-5p positively regulates the expression of the *NFE2L2* gene in PTC. This study showed the important relationship between miR-17-5p and *NFE2L2*, evidenced by the functional experiments. However, further studies regarding the influence of miR17-5p on *NFE2L2* and *VEGFA* and on the formation of new vessels in the colloid goiter and in PTC are needed. 

## Figures and Tables

**Table 1:** Characteristics of the collected samples.

Characteristics	Tumor	Goiter
**Gender**		
Female (F)	13 (86.7%)	14 (93.4%)
Male (M)	2 (13.3%)	1 (6.6%)
**Age**		
<45	F: 7 (46.6%); M: 1 (6.7%)	F: 6 (40%); M: 1 (6.7%)
≥45	F: 6 (40%); M: 1 (6.7%)	F: 8 (53.3%); M: 0 (-)
**Tumor extent**		
I	8 (53.4%)	
II-III	7 (46.6%)	
**Nodal metastasis**	2 (13.3%)	
**Distant metastasis**	2 (13.3%)	

**Table 2:** Genes and miR expression in thyroid tumor and goiter in relation to normal thyroid tissue.

Tumor					Goiter			
Gene	RQ Median	Min	Max	*P*	RQ Median	Min	Max	*P*
*VEGFA*	1.516	0.059	6.605	0.0125 *	20.010	8.595	32.260	<0.0001 *
*NFE2L2*	5.446	0.045	40.76	0.0061 *	23.380	0.278	68.780	0.0009 *
**MicroRNAs**								
miR-17-5p	0.206	0.007	3.305	0.094	0.099	0.006	0.879	<0.0001 *
miR-612	0.181	0.002	7.097	0.135	0.044	0.003	0.238	0.015 *

RQ, relative quantification; *P, p* value; *, Wilcoxon signed rank test.

**Table 3:** Gene and miR expression in tumor- and goiter-adjacent tissues in relation to normal thyroid tissue.

Tumor-Adjacent Tissue					Goiter -Adjacent Tissue			
Gene	RQ Median	Min	Max	*P*	RQ Median	Min	Max	*P*
*VEGFA*	3.405	0.010	8.190	0.0023 *	20.720	13.820	55.970	<0.0001 *
*NFE2L2*	23.990	0.039	76.920	0.0149 *	15.870	2.417	83.740	<0.0001 *
**MicroRNAs**								
miR-17-5p	0.256	0.059	11.020	0.118	0.209	0.043	10.930	0.0448 *
miR-612	0.128	0.003	20.790	0.016 *	0.092	0.001	4.413	0.0131 *

RQ, relative quantification; *P*, *p* value; *, Spearman correlation.

**Table 4:** Correlation between expression levels of *VEGFA* and *NFE2L2* and the miR-17-5p and miR-612 miRs in thyroid tumors and colloid goiter.

	Tumor				Goiter			
	*VEGFA*		*NFE2L2*		*VEGFA*		*NFE2L2*	
	R^2^	*P*	R^2^	*P*	R^2^	*P*	R^2^	*P*
**miR17-5p**	−0.411	0.130	−0.067	0.019 *	−0.118	0.653	−0.174	0.503
**miR-612**	−0.546	0.038 *	−0.679	0.007 *	-0.479	0.062	−0.724	0.002 *

R^2^, correlation coefficient; *P*, *p* value; *, Spearman correlation.

## Supplementary Materials

The following are available online at https://www.mdpi.com/2073-4425/11/9/954/s1, Figure S1: Expression of *VEGFA* in THCA based on Sample types; Figure S2: Expression of *VEGFA* in THCA based on Tumor histology; Figure S3: Expression of *NFE2L2* in THCA based on Sample types; Figure S4: Expression of *NFE2L2* in THCA based on Tumor histology; Table S1: *P* values for *VEGFA* gene comparisons; Table S2: *P* values for *NFE2L2* gene comparisons
