# Supplementary material for: VEGFA and NFE2L2 Gene Expression and Regulation by MicroRNAs in Thyroid Papillary Cancer and Colloid Goiter
Source: Genes (Basel). 2020 Aug 19;11(9):954. doi: 10.3390/genes11090954 (PMC7563674; doi:10.3390/genes11090954)
Supplement: Supplementary file 1 [file genes-11-00954-s001.pdf]

Supplementary material

The TCGA thyroid cancer dataset of genes VEGFA and NFE2L2.

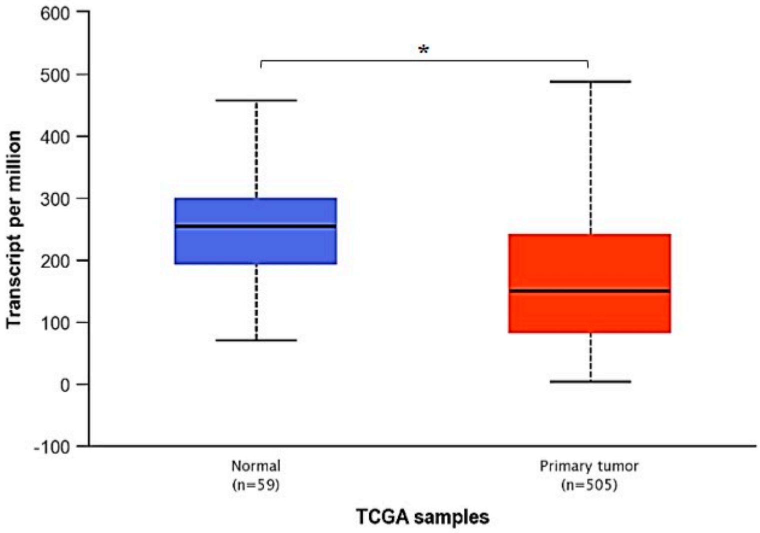

Figure 1. Expression of *VEGFA* in THCA based on Sample types

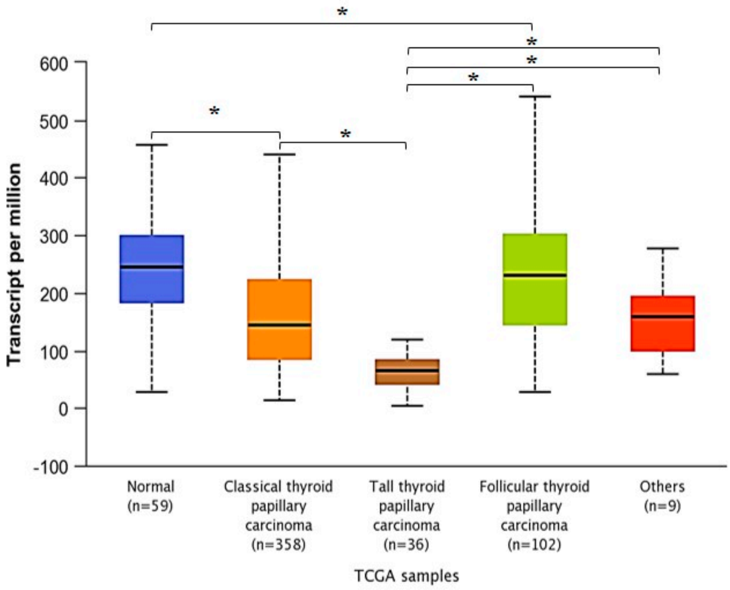

Figure 2. Expression of *VEGFA* in THCA based on Tumor histology

Tabela 1 – P values for *VEGFA* gene comparisons.

Comparison	Statistical significance
**Normal-vs-Primary**	**2,82E+07***
**Classical-VS-Tall**	**9,71E+00***
**Classical-VS-Follicular**	**8,74E+07***
Classical-VS-Other	7,64E+05
**Classical-VS-Normal**	**6,28E+07***
**Tall-VS-Follicular**	**1,62E+02***
**Tall-VS-Other**	**2,43E+04***
**Tall-VS-Normal**	**1,62E+02***
Follicular-VS-Other	1,85E+05
Follicular-VS-Normal	9,52E+05
Other-VS-Normal	5,88E+04

Figure 3. Expression of *NFE2L2* in THCA based on Sample types

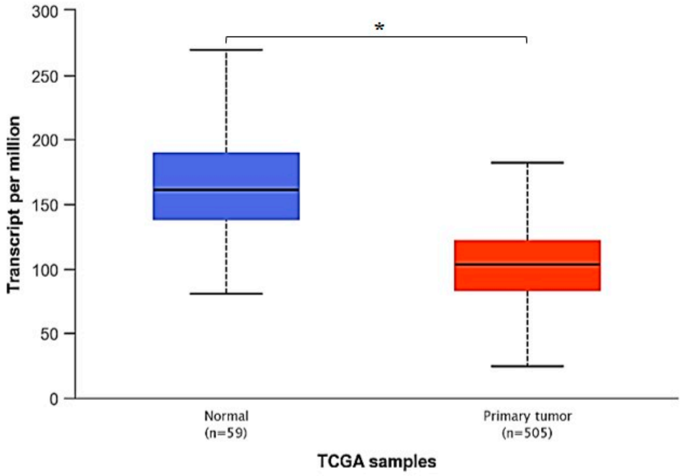

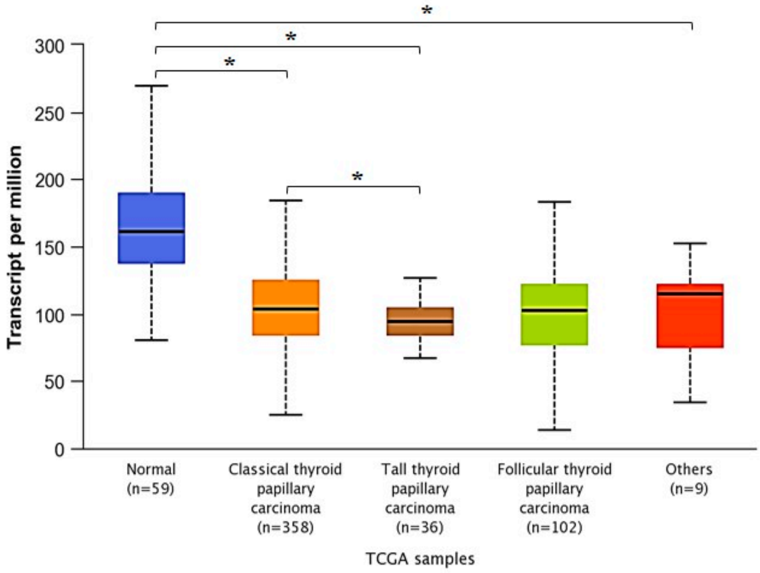

Figure 4. Expression of *NFE2L2* in THCA based on Tumor histology

Tabela 2 – P values for *NFE2L2* gene comparisons.

Comparison	Statistical significance
**Normal-vs-Primary**	**5,44E+06***
**Classical-VS-Tall**	**4,12E+03***
Classical-VS-Follicular	3,90E+05
Classical-VS-Other	6,11E+05
**Classical-VS-Normal**	**4,69E+02***
Tall-VS-Follicular	2,21E+05
Tall-VS-Other	4,89E+05
**Tall-VS-Normal**	**1,64E+02***
Follicular-VS-Other	3,31E+05
Follicular-VS-Normal	4,97E+05
**Other-VS-Normal**	**7,20E+02***
